# Changes in electrophysiological findings of spinal muscular atrophy type I after the administration of nusinersen and onasemnogene abeparvovec: two case reports

**DOI:** 10.1186/s12883-023-03420-2

**Published:** 2023-10-31

**Authors:** Tomoko Mizuno, Tadashi Kanouchi, Yumie Tamura, Ko Hirata, Runa Emoto, Tomonori Suzuki, Kenichi Kashimada, Tomohiro Morio

**Affiliations:** 1 Department of Pediatrics and Developmental Biology, Tokyo Medical and Dental University, 1- 5-45, Yushima, Bunkyo-ku, Tokyo, 113-8519 Japan; 2 Department of Laboratory Medicine, Tokyo Medical and Dental University, 1-5-45, Yushima, Bunkyo-ku, Tokyo, 113-8519 Japan

**Keywords:** Spinal muscular atrophy, Nusinersen, Onasemnogene abeparvovec, Nerve conduction study, Needle electromyography

## Abstract

**Background:**

Recently, there have been significant advances in the treatment of spinal muscular atrophy (SMA). Although clinical improvement in patients with SMA after the treatment has been reported, changes in electrophysiological findings, especially needle electromyography (EMG), have rarely been reported. Herein, we report the posttreatment changes in EMG and nerve conduction study findings over time in two patients with SMA type I.

**Case presentation:**

Patient 1: A 2.5-year-old girl was diagnosed with SMA type I at 1 month of age. She received nusinersen four times and onasemnogene abeparvovec (OA) was administered at 6 months of age. The compound muscle action potential (CMAP) amplitudes of the median and tibial nerves increased over time. The needle EMG after the treatment showed high-amplitude motor unit potentials (MUPs) suggestive of reinnervation during voluntary contraction, which were not seen before the treatment. However, fibrillation potentials at rest were still seen after the treatment. Patient 2: A 2-year-old girl was diagnosed with SMA type I at 6 months of age. She had received nusinersen two times and OA was administered at 7 months of age. The CMAP amplitudes and the MUPs presented similar changes as presented in Case 1.

**Conclusion:**

This is the first report on the changes in needle EMG findings after treatment in patients with SMA type I. These findings suggested that peripheral nerve reinnervation occurred after the treatment, although active denervation was still present. The accumulation of these findings will be important for evaluating the effectiveness of treatment for SMA in the future.

## Background

Spinal muscular atrophy (SMA) is an autosomal recessive neurodegenerative disorder caused by deletions or variants in the survival motor neuron 1 (SMN1) gene, and approximately 95% of all patients with this condition have a homozygous *SMN1* deletion [1, 2]. It causes motor neuron degeneration and progressive muscle weakness and atrophy, resulting in significant motor disability, respiratory and swallowing dysfunction. It is classified into types 1 through 4 based on the age of onset, symptoms, and maximum motor milestones achieved [3]. Patients diagnosed with SMA type 1, known as Werdnig–Hoffmann disease, display symptoms in the first 6 months of infancy, and never achieve the ability to sit independently. The neighboring *SMN2* can, in part, compensate for nonfunctional *SMN1*; hence, higher *SMN2* copy numbers in SMA are associated with later onset and milder phenotypes [4, 5].

Recently, there have been significant advances in the therapeutic field, and three different drugs have been approved [6]. Nusinersen, an antisense oligonucleotide drug, and risdiplam, a small-molecule drug, modify the pre-mRNA splicing of *SMN2* to increase the production of functional SMN, albeit by different mechanisms [7, 8]. The other therapy known as onasemnogene abeparvovec (OA) is a form of adeno-associated virus serotype 9 vector-based gene therapy delivering a transgene encoding human SMN proteins [9, 10]. Although clinical improvement in patients with SMA after these treatments has been reported [7–10], changes in electrophysiological findings have rarely been reported. Compound muscle action potential (CMAP) amplitudes in nerve conduction study (NCS) of patients with SMA type I treated with nusinersen or OA have been reported to increase over time compared to nontreated patients [7, 9]. However, changes in needle electromyography (EMG) findings after these treatments have not yet been reported. In this paper, we report changes in NCS and needle EMG findings over time in two cases with SMA type I after treatment with nusinersen and OA.

## Case presentation

### Patient 1

The proband was born to nonconsanguineous parents at 37 weeks of gestation. She was noted to have muscle hypotonia during the 1-month medical checkup appointment and was referred to our hospital. Her physical examination revealed muscle hypotonia and weakness, wrist drop, tongue fasciculations, and the absence of limb tendon reflex. She was able to lift only her forearm against gravity, but unable to lift her upper arms and lower limbs. Multiplex ligation-dependent probe amplification of the *SMN* showed homo deletions of *SMN1* exons 7 and 8 and two copies of the *SMN2*; thus, she was diagnosed with SMA type I. She had received nusinersen four times since she was 2 months old, and OA was administered at 6 months of age. After the treatment, she got to be able to lift her entire upper limbs against gravity first compared to her lower limbs. Children’s Hospital of Philadelphia Infant Test of Neuromuscular Disorders (CHOP-INTEND) score was 18 at 2 months (before nusinersen started), 37 at 6 months (before OA was administered), 46 at 1 year (6 months after OA), and 58 at 2 years (18 months after OA). She acquired head control at 10 months, sitting without support at 1 year, and rolling over at 1 year and 7 months. She is now 2 and a half years old and training to stand using a long leg brace (Table 1). She has had no problems with respiratory or swallowing function.

**Table 1:** CHOP-INTEND score, motor development, and CMAP amplitudes of the median and tibial nerves in two patients

**Patient 1**		**2 months old** **(before nusinersen started)**	**1 year old** **(6 months after OA)**	**2 years old** **(18 months after OA)**
CHOP-INTEND score		18	46	58
motor ability			head control sitting	rolling over
CMAP	median nerve	0.60 mV (-3.4 SD)	1.60 mV (-2.2 SD)	2.61 mV (-2.7 SD)
	tibial nerve	0.60 mV (-9.9 SD)	4.97 mV (-2.0 SD)	7.33 mV (-1.2 SD)
**Patient 2**		**6 months old** **(before nusinersen started)**	**1 year old** **(5 months after OA)**	**2 years old** **(17 months after OA)**
CHOP-INTEND score		15	43	49
motor ability				no head control rolling to side
CMAP	median nerve	0.26 mV (-2.3 SD)	2.31 mV (-1.8 SD)	2.56 mV (-2.7 SD)
	tibial nerve	1.11 mV (-3.2 SD)	1.21 mV (-3.3 SD)	3.86 mV (-2.3 SD)

Patient 1 received nusinersen four times since she was 2 months old, and OA was administered at 6 months old.

Patient 2 received nusinersen two times since she was 6 months old, and OA was administered at 7 months old.

SD values are calculated from the mean and SD values of healthy children at each age [13].

CHOP-INTEND, Children’s Hospital of Philadelphia Infant Test of Neuromuscular Disorders; CMAP, compound muscle action potential; OA, onasemnogene abeparvovec

**Fig. 1:**
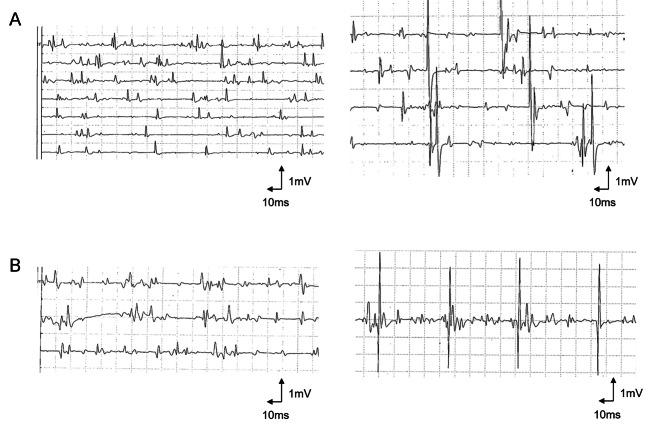
The needle EMG findings in the deltoid muscles during voluntary weak contractions in two patients 1a: In patient 1, the amplitudes of the MUPs were mostly around 0.5mV at 2 months of age (left), but the MUPs at 1 year of age showed very high amplitude, reaching 7–8 mV (right) 1b: In patient 2, the amplitudes of the MUPs were mostly around 0.5mV at 6 months of age (left), but the MUPs at 1 year of age showed very high amplitude, reaching 6–7 mV (right)

In NCS, motor nerve responses were evoked under intravenous sodium thiopental-induced sleep, while the skin temperature was kept higher than 31 °C. In needle EMG, a needle was inserted into deltoid and rectus femoris muscles during intravenous sodium thiopental-induced sleep. After recording at rest, we awakened the patient and recorded the examination of voluntary contraction. The CMAP amplitudes of the median and tibial motor nerves at 2 months, 1 year, and 2 years of age are shown in Table 1. Although the CMAP amplitudes of both nerves were very low before nusinersen started, they increased over time after the treatment, and that of the tibial nerve reached the normal range 18 months after OA administration. The MCVs of these nerves were always in the normal range. The F-wave evaluation was not performed because she was sedated. The needle EMG showed fibrillation potentials at rest in both the deltoid and rectus femoris muscles at 2 months, 1 year, and 2 years of age. During voluntary weak contractions, the amplitudes of motor unit potentials (MUPs) were mostly around 0.5 mV at 2 months of age; however, the MUPs at 1 year and 2 years of age showed high amplitudes that reached 7–8 mV (Fig. 1a).

### Patient 2

The proband was born to nonconsanguineous parents at 37 weeks of gestation. Her parents observed that she had little limb movement. She was hospitalized at 6 months of age with atelectasis due to a common cold. She showed muscle hypotonia and weakness, and no antigravity movement of her upper and lower limbs. She also showed tongue fasciculations and paradoxical breathing. She was referred to our hospital and diagnosed with SMA type I, with homo deletions of *SMN1* exons 7 and 8, and two copies of the *SMN2*. She had received nusinersen twice and OA was administered at 7 months of age. The CHOP-INTEND score was 15 at 6 months (before nusinersen started), 16 at 7 months (before OA was administered), 43 at 1 year (5 months after OA), and 49 at 2 years (17 months after OA) (Table 1). She revealed poor oxygenation at night at 1 year and 2 months of age and noninvasive intermittent positive pressure ventilation was introduced. She had been fed through a gastric tube since she was 6 months old; however, she was able to take oral nutrition fully at 1 and a half years old. She has acquired rolling to side, but not acquired head control at her current age of 2 years.

NCS and needle EMG were performed in the same manner as in patient 1. The CMAP amplitudes of the median and tibial nerves at 6 months, 1 year, and 2 years of age are shown in Table 1. The MCVs of these nerves were always in the normal range. The F-wave evaluation was not done because she was sedated. Needle EMG revealed her fibrillation potential at rest in both the deltoid and rectus femoris muscles at 6 months and 1 year of age. During voluntary weak contractions, the amplitudes of the MUPs were mostly around 0.5 mV at 6 months; however, the MUPs at 1 year revealed a high amplitude that reached 6–7 mV (Fig. 1b).

Informed consent was obtained from the parents of each patient for the use of their child’s data.

## Discussion and conclusion

We report changes in NCS and needle EMG findings over time in two cases with SMA type I treated with nusinersen and OA in this paper. NCS in patients with SMA typically shows decreased CMAP amplitudes reflecting chronic motor axonal degeneration, and CMAP amplitudes correlated with clinical severity and age [2, 11, 12]. The peroneal CMAP amplitude was reported to increase in infantile-onset SMA treated with nusinersen compared to the nontreated SMA [7]; the same has been reported for the ulnar CMAP in SMA treated with OA [9]. In our patients, the CMAP amplitudes of the median and tibial motor nerves were significantly lower than those of healthy children of the same age before the treatment [13]. However, they increased over time after the treatment and some of them reached the normal range. Previous papers have reported that patients with SMA gain better motor development, the earlier they are treated [7], and that patients with symptomatic SMA who received nusinersen showed a significant increase in CMAP and motor unit number estimation was more increased in patients with shorter disease durations [14]. The treatment was initiated earlier in patient 1 (2 months old) than in patient 2 (6 months old), so patient 1 probably had better acquisition of motor development and more elevated CMAP amplitude of the tibial nerve. In patient 1, the CMAP amplitude of the tibial nerve had better recovery compared to the median nerve. However, clinical improvement was greater in the upper limbs, so the difference in the degree of improvement in CMAP between upper and lower limbs may not necessarily reflect the degree of clinical improvement.

In contrast, the changes in needle EMG findings after these therapeutic interventions have not yet been reported. Needle EMG in patients with SMA reveals abnormal spontaneous activity, including fibrillation potentials at rest reflecting active denervation and long durations and high amplitudes of MUPs during voluntary contraction reflecting chronic compensatory changes of reinnervation. However, in severely affected patients like SMA type I, MUPs may reveal reduced amplitudes and durations because of severe active denervation [2]. McDonald described that MUPs were lower in infants with amplitudes ranging from 150 µV to approximately 2000 µV, and generally MUPs more than 1000 µV in 0–3-year-old children are rare [15]. In our patients, needle EMG after the treatment showed high-amplitude MUPs that reached 6–8 mV during voluntary weak contractions, which were not seen before the treatment. It is known that high-amplitude MUPs are seen when nerve reinnervation occurs after denervation [16]; so, it is suggested that peripheral nerve reinnervation occurs after these treatments, but it does not necessarily prove recovery of motor neurons. In fact, fibrillation potentials suggestive of denervation were still seen in both patients. There are no reports on the changes in needle EMG findings in patients with SMA after the treatment; so, we need to follow how these reinnervation and denervation findings change. The accumulation of these findings will be important for evaluating the effectiveness of treatment for SMA in the future.

## Data Availability

The data that support the findings of this report are available from the corresponding author, TM, upon reasonable request.
